# A chemist and biologist talk to each other about caged neurotransmitters

**DOI:** 10.3762/bjoc.9.8

**Published:** 2013-01-11

**Authors:** Graham CR Ellis-Davies

**Affiliations:** 1 Department of Neuroscience, Mount Sinai School of Medicine, One Gustave Levy Place, New York, NY 10029, USA

**Keywords:** caged compounds, cell signaling, electrophysiology, neuronal currents, photolabile neurotransmitters, rates of reaction, receptor antagonism

## Abstract

Caged compounds are small organic molecules that can be photoactivated with brief pulses of light. They are widely used to study a great variety of biological processes by physiologists, cell biologists and neuroscientists. Initially made and invented by biologists in the late 1970s, they are now made mostly by chemists, often without any dialogue with the end users, the biologists. The idea for this review is to stimulate interaction between the two communities to further the creative development and application of these powerful optical probes.

## Introduction

The first biologically active molecules to be synthesized with photochemically protecting groups at their active sites were nucleotides [1–2]. Two reports appeared in 1977 and 1978 describing the synthesis of ortho-nitrobenzyl derivatives of cyclic-AMP [1] and ATP [2]. The photolabile cAMP derivative was one member of a series of phosphate esters made as membrane permeable pronucleotides. Thus, this optical probe arose out of the context of the already developed prodrugs that used thermal chemistry for release of their latent cargo. In contrast, the photolabile ATP molecule was synthesized in a physiology department for rapid photoactivation of a particular enzyme, the Na,K-ATPase. It was the latter group that dubbed such photochemical probes “caged compounds”. This simple term has been adopted by biologists since that time [3–9], perhaps because the photolabile ATP compound was the one that was used in a series of important muscle physiology studies in the 1980s [6,10–13]. Chemists have been much more resistant to embrace the moniker, mostly because the term is used for cagelike structures (e.g., cubane), but also perhaps due to the “lateness of arrival” into the field, which gave rise to attempts to commandeer the field for themselves by using a host of different terms [14–16]. Having expressed this opinion, there is no doubt that chemists were slow to use their great synthetic skills to help biologists develop new caged compounds. It can be seen that in the 1980s all the important new caged compounds were made in biology departments: (1) caged calcium [17] (molecular biology), (2) caged IP3 [5] (physiology) and carbamoylcholine [9] (biochemistry). The last of these new probes was the beginning of the development of caged neurotransmitters (e.g., glutamate, GABA, sertonin, glycine) and an important collaboration [18–24] between George Hess (a biophysicist) and Barry Carpenter (a chemist).

Why was this collaboration important for this field? The answer has two parts. First, it is rare for one group to encompass all the expertise required for the development of such optical probes, as skills in organic chemistry, photophysics, ion-channel biophysics and neurobiology are ideally all involved in the process. Secondly, the main goal of the team was set by the biological problem [25], and often such problems are not well understood by chemists working in isolation. Thus, to solve the biological problem provided by the biologist (Hess), a newer caging chromophore [18] had to be synthesized by a chemist (Carpenter). Thus, chemists came to play a crucial role in the caged compound field. Chemistry at the service of biology is not unusual or unique, as the pharmaceutical industry is built on this idea. In my view it still remains difficult for organic chemists and neurobiologists to understand the limitations of the others’ field, and it is these limitations that are crucial for compound development. In this review I try to take the side of the neurobiologist who is thinking about using a caged transmitter in an experiment. I ask the chemist a series of simple questions about the caged compound(s); some of these questions, we discover, do not have simple answers.

**Figure 1:**
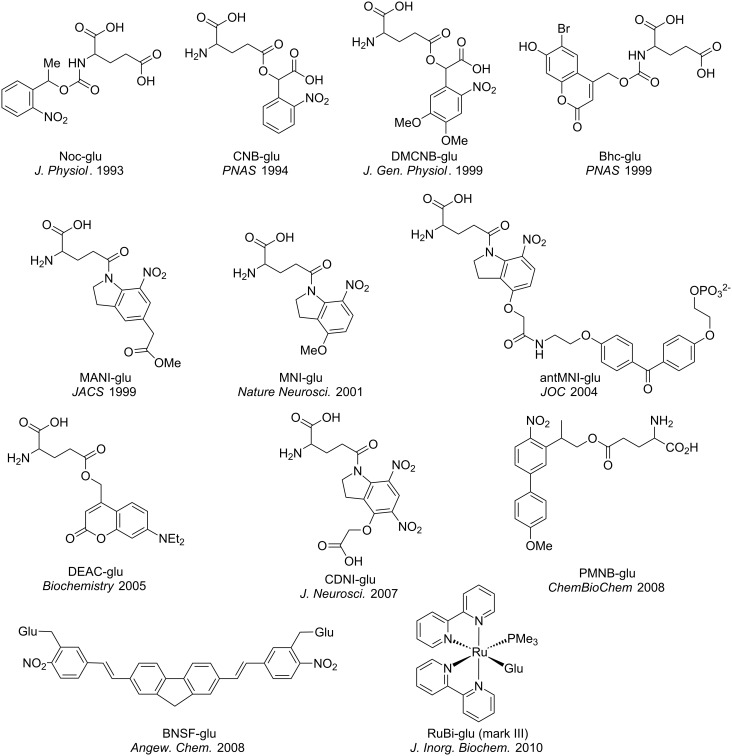
Structures of various caging chromophores. Abbreviations: Noc, *N*-nitrophenethyloxycarbonyl; CNB, carboxynitrobenzyl; DMCNB, dimethoxycarboxynitrobenzyl; Bhc, bromohydroxycoumarin; MANI, methylacetoxynitroindolinyl; MNI, methoxynitroindolinyl; antMNI, antennea-methoxynitroindolinyl; DEAC, diethylaminocoumarin; CDNI, carboxymethylnitroindolinyl; PMNB, propylmethoxynitrobiphenyl; BNSF, bisnitropropylstyrylfluorene; RuBi, ruthenium-bipyridine.

Glutamate is the most important neurotransmitter in the brain, as its release at nerve synapses transfers electrical signals between pre- and postsynaptic cells. Approximately 80% of such signaling is carried out by glutamate binding to AMPA receptors, the major post-synaptic excitatory ionotropic ion channels that bind glutamate. These receptors are blocked specifically by α-amino-3-hydroxy-5-methyl-4-isoxazolepropionic acid, hence their acronym. For this reason the development of caged forms of this neurotransmitter has been the subject of considerable activity by chemists. Furthermore, the vast majority of neurons and all astrocytes have glutamate receptors involved in other types of cellular signaling, and so many biologists are interested in stimulating these receptors. I focus the conversation on caged glutamate probes (Figure 1).

## Discussion

Biologist: Is your caged glutamate "biologically inert"?

Chemist: We have several caged glutamate molecules [7,23,26–34], and some are even commercially available [18,26–27,31,33] (Table 1). In order to address your question, we can see that in most of the original papers describing the development of the compounds, detailed pharmacology was described. In one report, the spontaneous miniature excitatory post-synaptic currents (EPSCs) were measured in the presence and absence of a probe when it was puffed at 10 mM concentration onto cultured hippocampal neurons [31]. The histograms of the ensemble averages were indistinguishable. The other examined whether MNI-Glu blocked the current evoked by puffer application of glutamate itself onto neurons [35]. Since these original reports, many other groups have reproduced these results, so they are probably reliable. A recently developed caged glutamate that uses different photochemistry ("RuBi-Glu") is also reported to be inert towards AMPA receptors [33]. This study also noted that MNI-Glu perturbed AMPA spontaneous miniature EPSCs. So it is difficult for us chemists to judge from such reports how to improve the caged compounds any further. In fact, in our own field we know very well, that the same reagent can give different results under slightly different conditions, or even the same conditions!

**Table 1:** Properties of various caged glutamate probes^a^.

Caged Glu	ε (λ_max_)	Φ (% Glu yield)	ε·Φ	2PuCS (GM/nm)	Commercial	Pharmacology towards GABA-A	Stability in aqueous buffer	Solubility (mM) at pH 7.4 in aqueous buffer

Noc	500 (350)	0.65 (100)	325	NR	none	NR	Stable	>50
CNB	500 (350)	0.14 (100)	60	NR	Invitrogen	Partial agonist	Half-life 17 h rt	>50
MNI	4,300 (330)	0.085 (>95)	357	0.06 (740)	Tocris	SA 10 mM	Stable	400
RuBi	5,600 (450)	0.13 (NQ)	728	0.14 (800)	Tocris	50% inhibition at 0.3 mM	Stable	NR
PMNB	9,900 (317)	0.1 (100)	990	0.45 (800)	none	NR	Stable	Requires 1% DMSO
antMNI	27,000 (300)	0.085 (94)	2295	NR	none	NR	Stable	33
BNSF	64,000 (415)	0.25 (65)	16,000	5 (800)	none	NR	ND	0.1
CDNI	6,400 (330)	0.6 (100)	3,840	0.06 (720)	none	MA 0.4 mM	Stable pH 2	100
DEAC	13,700 (390)	0.11 (NQ)	1507	NR	none	NR	stable	NR
MANI	4,300 (330)	0.1 (100)	430	NR	Sigma	NR	Like MNI	>100
Bhc	43,000 (458)	0.3 (100)	12,900	1 (740)	none	NR	Stable frozen pH 7.4	7.5

^a^Abbreviations and symbols: ε, extinction coefficient; Φ, quantum yield; 2PuCS, 2-photon uncaging cross section; NR, not reported; MA, mild agonist; Noc, *N*-nitrophenethyloxycarbonyl; CNB, carboxynitrobenzyl; MNI, methoxynitroindolinyl; RuBi, ruthenium-bipyridine; PMNB, propylmethoxynitrobiphenyl; antMNI, antennea-methoxynitroindolinyl; BNSF, bisnitropropylstyrylfluorene; CDNI, carboxymethylnitroindolinyl; DEAC, diethylaminocoumarin; MANI, methylacetoxynitroindolinyl; Bhc, bromohydroxycoumarin.

Biologist: Neuroscientists most often simply want the technology they buy to “work”. So it can be inferred that if many different groups have successively used some piece of technology, it is probably fine.

Chemist: We chemists have a similar appreciation of technology in our own field. For example, in the field of the development of reagents for synthetic methods, there are examples of reagents that have been found to give very reliable and reproducible yields for the transformation that they are designed to accomplish, by many laboratories. In contrast, the literature is full of "single-data-point reports" of chemical transformations. Naturally chemists tend to trust the former.

Biologist: It is well known from work in the pharmaceutical industry that drugs developed to target receptors can have undesirable and surprising side effects, so-called off-target effects. What about these caged Glu probes?

Chemist: It is much harder to give a clear-cut answer to this question. At the high concentrations that are used for two-photon experiments (range 3–12 mM) all classes of caged neurotransmitters are reported to block GABA-A receptors to some extent [33,36–37]. This off-target effect was only discovered relatively recently, as it never occurred to anyone that probes such as MNI-Glu, which were inert toward their target [31], would block another neuroreceptor in the same concentration range [36]. The structure–function relationship for such effects is not fully understood. What is known is that similarly caged Glu and GABA probes (e.g., CDNI-Glu and -GABA) both block GABA-A receptors with the same efficacy, implying that the amino acid is not crucial. Furthermore, the diversity of the structures of caging chromophores (Figure 1) implies that one specific functionality cannot give rise to such undesirable effects. However, at low concentrations (range 5–300 µM) it seems that these off-target effects are significantly reduced [38–39]. For other glutamate receptors, such as amino acid transporters involved in the clearance of glutamate by astrocytes, caged compounds do not block such receptors [40]. The effect of caged transmitters on other membrane receptors, such as voltage-caged ion channels, has not been well studied. The good news is that since the caged transmitters have a relatively low affinity for GABA-A receptors, it turns out that most, if not all, can be overcome by uncaging anyway [36]. Thus, I would like to ask, how important are such off-target effects for your experiments?

Biologist: This is a good question. Perhaps we biologists simply don't like the idea of using probes that slightly perturb the system we study in some way, even if it does not make much difference to the question we are addressing. One type of experiment one could imagine performing where it may be important is the combination of two-photon uncaging of glutamate with optogenetic activation of GABA receptors from genetically selected neurons. In such a paradigm the application of high concentrations of caged Glu would probably block the effects of synaptically released GABA.

A possible advantage of blockade is that it may enhance the spatial restriction of photoreleased transmitter. Certainly, a smart experiment would be to take advantage in some way of the poor pharmacology of caged transmitters towards GABA receptors. Two good examples from another area are the known chloride and pH sensitivity of wild-type GFP fluorescence being used as indicators for these solutes [41–42]. Most fluorescent proteins we use have these properties mutated away; however, the genetically encoded indicators actually use the “weaknesses”. Some photolabile neurotransmitters have now been developed and are commercially available without any testing of any sort being performed. Thus, they cannot even really be called “caged”.

Biologist: Is your caged transmitter "water soluble"?

Chemist: It is well known in the pharmaceutical industry that water solubility is one of the true challenges for drug development. Often drugs are built up around rigid hydrocarbon scaffolds, such substances are inherently hydrophobic. The same issue applies to caged compounds, as in these we add aromatic rings to produce photosensitivity. The simplest type of caging chromophores are quite water soluble (Table 1), but even modest derivatives can lose water solubility precipitously! With drugs this is often counteracted by adding betacyclodextrin to the formulation, and with caged compounds, a small amount of organic solvent (ca. 1%) is what we chemists typically resort to [28,43–44], but sometimes much larger proportions are required for effective uncaging [45], rendering the method biologically useless. What is acceptable to, or ideal for neurobiologists?

Biologist: Last time I looked there was no organic solvent in the brain. The pH of physiological buffer is in the range of 7.2–7.4 and is set by dissolved CO_2_. For obvious reasons, neurobiologists who use acutely isolated brain slices to study neuronal function mimic this buffer. Neurons are the most sensitive cells, so even small amounts of organic solvents can be quite toxic, and thus no one would routinely use any DMSO or methanol in their buffer. Perhaps 0.1% is OK if one is desperate; but ideally no organic solvents should be present in the buffer.

Chemist: We chemists tend not to be too concerned about reagent concentrations in our work, as long as the substrates dissolve, we are fine. All caged neurotransmitters are water soluble to some extent, so how soluble do they have to be?

Biologist: We are very concerned about concentrations! But the exact amounts used always depend on the type of experiment. Routine one-photon uncaging only requires a concentration of 1 mM (maximally), and quite often, much lower concentrations work very well [46–49]. As we have already noted, two-photon uncaging normally requires significantly higher concentrations [30,50–53], so for these experiments solubility becomes more of an issue. It is important for chemists to know that we usually make up stock solutions that are at least 10 times higher than those used, therefore we would look for water solubility in the 50–100 mM range.

Chemist: This can easily be achieved for caged transmitters with simple chromophores such as MNI-Glu and CNB-Glu. But even the addition of one nitro group or aromatic ring to these molecules dramatically reduces their solubility properties making them much more difficult to use [34]. But note the new RuBi-Glu [33] probe is soluble in water up to 20 mM, even though it has many aromatic rings.

Biologist: How water stable are the caged neurotransmitters at physiological pH?

Chemist: Again this varies tremendously. The nature of the chemical bond used for caging defines the aqueous stability. Simple benzyl esters are fairly stable, so CNB-Glu [18] has a decent half-life at pH 7.4. However, more electron-rich molecules tend to be much more unstable. Phosphate esters are more stable than carboxylate esters, but they also suffer from instability. Thus, an electron-deficient NB-caged cAMP is very stable but an electron rich one (DMNB-cAMP) is not [4,54]. The opposite trend is true for nitroindolinyl-caged transmitters [26–27,32,36]. Because of this problem, chemists have resorted to inserting a spacer unit between the caging chromophore and the substrate [7,55–59]. This strategy has been very successful in creating some highly stable caged compounds, but sometimes at the expense of another property, such as the rate of release [7]. For example, GABA caged directly as an ester with coumarin chromophores is photoreleased quickly, but is quite unstable in (frozen) solution [60]. However, when caged via a carbamate, its release is orders of magnitude slower, but the compounds are water stable [56]. When phenols (e.g., serotonin or capsaicin) are caged via the carbonate, they are effectively stable and released quickly [61–62]. It is also important to note that the stability of all these “acid-like” caged compounds depends on the pH of the aqueous solution. All are more stable in the pH range of 2–4, and much less stable above pH 8. Thus, it may be better for long-term storage of solutions to be done at the former, with daily solutions diluted from stocks.

Other caged compounds, due to the chemical bond that is used, are impossible to hydrolyze. For example, all ethers and amines caged with nitrobenzyl groups are completely stable [18]. RuBi-Glu and GABA are also stable [33,38], as these are caged using donation from the amine lone pair of the neurotransmitter into the Ru d-orbital. Finally, it is important to note that many chemists do not study stability at physiological temperatures, nor do they perform long-term stability tests over many months. How important are such details for biological use?

Biologist: In fact this sort of information is really quite important. We often perform experiments with warm buffer (30–37 °C), or even in living animals. Also, for practical purposes we make stock solutions for freezing in aliquots, and only thaw for use at the desired time.

Chemist: Since 1980, the rate of release of the caged compound has been the subject of study by those who develop caged compounds [3]. Since the reaction mechanisms are quite complex, these details have also fascinated many chemists in the field of caged compounds [63]. In fact, we can get quite “distracted” by such arcane studies. Can you help with guidelines for the requirements you have for rates of uncaging?

Biologist: As you noted for chemical stability, such requirements vary a good deal! Our requirements are conditioned by two concerns. First, we are limited by our measurement ability. In terms of imaging or electrophysiology it is very difficult for us to measure anything in a cell faster than a few microseconds. In electrophysiology, we normally apply a digital filter to the signal. These are in the range of a few kilohertz, and such signals would be digitized at tens of kilohertz [64–65]. Imaging is slightly different but it is also relatively slow, and is defined by the ability to collect enough photons from a unit area (a pixel). For standard confocal imaging the dwell time for each pixel is a few microseconds, meaning an image frame takes about 1–2 seconds [66]. This is much slower than electrophysiology. There are several methods that are used to speed up the rate of image acquisition [67–68], but even these are limited to 30–100 Hz for full frame (512 × 512 pixels) imaging. Of course if one takes smaller frame sizes the rate increases. The most widely used method is simply eliminating the frame, by using “line-scan” imaging, in which each line may require only 2 ms. Some modern imaging software allows many “short lines” to be connected, enabling quite fast imaging of selected cellular areas [69–70].

Chemist: So if we uncage in the pico- or nanosecond time range [71–72] is this of any real use for biology?

Biologist: Not really. As mentioned above, because we are constrained by measurement protocols, probably the 1–10 microseconds range is sufficient for most purposes. I have been confused by some reports of “rates of reactions” being given as a time course for steady-state photolysis over many seconds or minutes [45], whereas other reports show rates from laser flash photolysis [3,61]. Can you help clarify this?

Chemist: Only the latter should be called a rate. The former is simply a way of measuring the quantum yield by determining the half-time (hence “rate”) of photolysis under some set of defined irradiation conditions. However, even with the former measurement care must be taken. The chemical reactions of release can be quite complex, so the rate of release of product is the key property [3,62–63]. In fact this is one reason why chemists study reaction mechanisms, as there can be several intermediates along the reaction pathway, leading to transmitter release [73]. Unfortunately, there is no easy way to detect glutamate optically, so the other products from uncaging are used as surrogates for the transmitter [61]. For example, protons can often be a reaction product, so pH detection is used [7–8]. If the aromatic side product has a distinctive absorption, the appearance of this species can be measured [3]. However, not all caged compounds have such side products, or their appearance may not be in the rate-limiting step along the reaction pathway.

Biologist: From receptor kinetics in membrane patches from cultured neurons, the estimated half-time of the increase in glutamate concentration is fast [74] (ca. 0.1 ms). The measured postsynaptic rise (10–90%) for excitation [75] is about 0.15–0.70 ms and inhibition [76] about 0.4 ms.

Chemist: Short periods of two-photon excitation (0.05 ms) allow one to mimick such events [31], implying the rate of uncaging of glutamate from MNI-Glu is not rate-limiting for optical stimulation of postsynaptic ionoropic receptors. RuBi-Glu is uncaged in less than 50 ns [77], so this probe may be used with similar confidence. The first fast caged glutamate, CNB-Glu, is photoreleased with a half-time of about 0.03 ms [18].

Chemist: A somewhat neglected property of caged neurotransmitters is their compatibility with biological buffers. This is a different issue from chemical stability and pure solubility. It seems that some phosphate derivatives of polyaromatic chromophores have been reported to precipitate in artificial cerebral spinal fluid [78–79]. This property has not been studied for many other caged transmitters, but probably the lack of difficulties for the most widely used caged compounds suggests that they are well tolerated in physiological buffers.

Biologist: What is the difference between photochemical and chemical efficiency for uncaging reactions?

Chemist: The photochemical efficiency of uncaging involves two completely distinct properties [80]. First, we must consider how well a molecule absorbs light, through the molar extinction coefficient, ε. This property measures how effectively a chromophore absorbs photons. Thus, fluorescein (ε = 80,000/M/cm) absorbs light much better than MNI-Glu [27] (ε = 4,300/M/cm). The second property is the quantum yield of photolysis (chemists use the symbol Φ for this). This measures how many excited molecules give a product, with the normal maximum being 1. Photochemical efficiency is ε·Φ. So, to some extent, a large ε can make up for a poor Φ, but ideally the Φ should be large, to take maximum advantage of the absorbed light. A large ε allows the use of less light, thus potentially avoiding phototoxic side effects from uncaging. However, it has been pointed out that very large ε are not always advantageous for 1-photon uncaging experiments [80–81]. If a solution of 1 mM is applied to a cell and the chromophore has an ε = 4,300/(M·cm), then a 1 mm path length absorbs a fraction 1 − log 0.43, and a 4 mm path length absorbs 1 − log 1.92. Such path lengths are typical for microscope objectives, meaning that much of the light will be absorbed before it reaches the cell [81]. This saturation problem is not an issue for two-photon excitation of caged compounds.

Chemical efficiency is unrelated to ε and Φ. It refers to the basic property of the chemical yield of glutamate compared to the amount of caged glutamate photolyzed. The good news is that most uncaging reactions are quite efficient chemically, with few competing side reactions [80]. One of the caging chromophores has been reported [29] to undergo a significant side reaction that traps the caged compound in a cul-de-sac. However, no reaction is completely “clean”, so it is doubtful that 100% release of glutamate can ever come from uncaging.

Biologist: Many really important biological experiments with caged transmitters have been performed with compounds that have relatively poor photochemical efficiency [82]. So why do you chemists continue to make new compounds if the old ones work?

Chemist: This is a good question. Certainly CNB-Glu [18] and CNB-GABA [83] yielded many useful results [82]. For one-photon uncaging the ε·Φ of these is sufficient. However, they are prone to hydrolysis, so the first nitroindolinyl-caged Glu was a real improvement, as it is much more stable at physiological pH [26]. Thus, for all 1-photon experiments this caged Glu compound is sufficient; however, it is not very sensitive to two-photon excitation. Hence, several other nitroindolinyl-caged transmitters have been made and used [31–32,34,36,39–40,56,84–85]. Since these have been designed for 2-photon uncaging, they have proved useful in many neurophysiological experiments in brain slices, especially those concerning the biochemistry in single spine heads. Note that MNI-Glu was independently made by two groups at the same time [27,86], but only one of them appreciated that the molecule would be useful for this area of optical neuroscience [31]. In reality, I think we chemists need guidance from you biologists as to what is required for the “next phase” of caged transmitter development.

**Figure 2:**
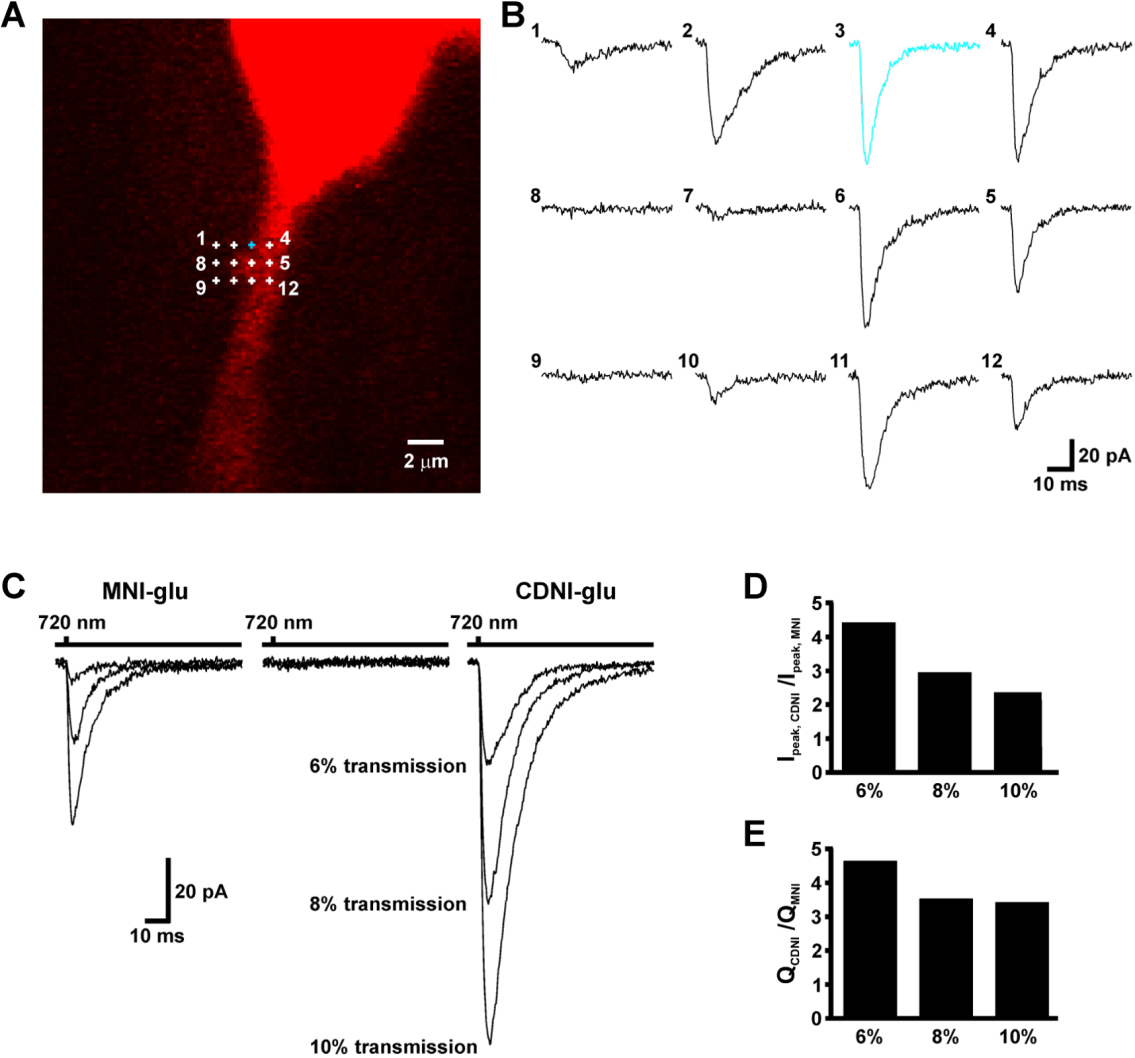
Comparative two-photon uncaging of MNI-Glu and CDNI-Glu on pyramidal neurons in an acutely isolated brain slice. (A) Fluorescent image of a pyramidal neuron filled with red dye. Numbered crosses correspond to the positions of the points of uncaging in B. (B) Currents evoked by two-photon irradiation at points marked in A. (C) Comparative photolysis of MNI-Glu and CDNI-Glu on the same neuron. The caged compound was topically applied from a puffer pipette at a concentration of 10 mM above the brain slice. Current traces are an average of several trials. (D, E) Relative peak currents and charges evoked by CDNI/MNI. Note some receptor saturation from uncaging the more photoactive CDNI decreases the ratio at higher powers. Data courtesy of Martin Paukert and Dwight Bergles (Johns Hopkins School of Medicine). The two caged compounds were applied "blind" during these experiments.

Biologist: Certainly one of the things that would be very helpful for us is that if you make a new caged transmitter, you perform some side-by-side real-world comparison. For example, when the genetically encoded calcium indicators (GECI) are made, it is standard practice to bench-mark them against calcium dyes and earlier GECI [87–93]. The other GE technology based on proteins called “channelrhodopsins” is also rapidly evolving, and this is done in a comparative way [94]. Thus, similar comparisons for each new caged neurotransmitter would be useful. Since one such example has been reported [32] (Figure 2), and several are now commercially available (Table 1) this seems a fairly straightforward task.

Chemist: Since the discovery of the light-gated ion channels called channelrhodopsins (ChR) in 2002 [95] and 2003 [96], a second method for optical activation of neuronal cells has been developed. Some think that the massive and expanding popularity of optogenetic stimulation shows that synthetic caged compounds remain a fairly mature niche technology. What do you think is required from chemists to sustain support for uncaging technology?

Biologist: First, it is important to say the obvious, that very clearly channelrhodopsin and caged compounds are complementary methods. But certainly many neuroscientists are using channelrhodopsin who did not use caged compounds. This is because they want to control the behavior of a moving animal [97–98]. Importantly, genetic methods allow ChR to be expressed in cells without any additional cofactors (no chemicals) and to select which cells have the ChR [94,98]. These two properties are very powerful advantages for neuroscience, compared to caged compounds. However, when it comes to other organs, where a firing action potential is not so important, it is not so clear how useful ChR can really be. Secondly, optogenetic control technology is limited to a few signaling cascade molecules [94], whereas caged compounds can be used for virtually any type of molecule [80]. However, all too often the ingenious inventions of organic chemists in this realm do not move beyond the initial proof-of-principle deployment [99–102]. Even in the case of caged glutamate compounds the vast majority of the new probes are not used to do new biological experiments (Table 1). Thus, I think it is vital for chemists to seek out closer collaborations with biologists of all sorts to enable the creative development of truly useful new caged compounds.

## Conclusion

Caged compounds have been uniquely powerful optically activated chemical tools for many areas of biological science. In particular, the past 20 years have witnessed an increased refinement in terms of the ability to localize neurotransmitter release. Starting from broad-scale mapping of synaptic connections, by using UV stimulation, to highly local concentration jumps, by two-photon excitation of caged glutamate compounds, caged glutamate probes continue to be widely used by the neuroscience research community. These probes could only have developed in the context of a fruitful dialogue between organic chemists and neurobiologists.
